# Engineering of a Promoter Repressed by a Light-Regulated Transcription Factor in *Escherichia coli*

**DOI:** 10.34133/2021/9857418

**Published:** 2021-09-28

**Authors:** Daniel Camsund, Alfonso Jaramillo, Peter Lindblad

**Affiliations:** ^1^Microbial Chemistry, Department of Chemistry-Ångström, Uppsala University, Uppsala, Sweden; ^2^Molecular Systems Biology, Department of Cell and Molecular Biology, Uppsala University, Uppsala, Sweden; ^3^Warwick Integrative Synthetic Biology Centre (WISB) and School of Life Sciences, University of Warwick, Coventry, UK; ^4^Génomique Métabolique, Genoscope, Institut François Jacob, CEA, CNRS, Univ Evry, Université Paris-Saclay, Evry, France; ^5^Institute for Integrative Systems Biology (I2SysBio), CSIC – Universitat de València, 46980 Paterna, Spain

## Abstract

Light-regulated gene expression systems allow controlling gene expression in space and time with high accuracy. Contrary to previous synthetic light sensors that incorporate two-component systems which require localization at the plasma membrane, soluble one-component repression systems provide several advantageous characteristics. Firstly, they are soluble and able to diffuse across the cytoplasm. Secondly, they are smaller and of lower complexity, enabling less taxing expression and optimization of fewer parts. Thirdly, repression through steric hindrance is a widespread regulation mechanism that does not require specific interaction with host factors, potentially enabling implementation in different organisms. Herein, we present the design of the synthetic promoter P*_EL_* that in combination with the light-regulated dimer EL222 constitutes a one-component repression system. Inspired by previously engineered synthetic promoters and the *Escherichia coli lacZYA* promoter, we designed P*_EL_* with two EL222 operators positioned to hinder RNA polymerase binding when EL222 is bound. P*_EL_* is repressed by EL222 under conditions of white light with a light-regulated repression ratio of five. Further, alternating conditions of darkness and light in cycles as short as one hour showed that repression is reversible. The design of the P*_EL_*-EL222 system herein presented could aid the design and implementation of analogous one-component optogenetic repression systems. Finally, we compare the P*_EL_*-EL222 system with similar systems and suggest general improvements that could optimize and extend the functionality of EL222-based as well as other one-component repression systems.

## 1. Introduction

Synthetic gene circuits in bacteria often rely on external inducers, and light offers the ability to regulate cellular processes with unsurpassed spatial and temporal resolution. Generally, exogenous wild-type or modified photoreceptors are heterologously expressed and then harnessed to control *in vivo* biological phenomena such as ion transport, protein interactions, or gene expression [1]. This versatility has led to great interest and diverse applications in, for instance, biomedical research or as characterization tools for synthetic and systems biology [2, 3]. In addition, we expect that light-regulated gene expression systems will have many applications in bacterial synthetic biology as alternatives to chemical effectors in general and, particularly, for gene regulation in solar-powered production hosts such as cyanobacteria [4, 5].

To date, most of the bacterial light-induced regulators are based on membrane-localized two-component systems [6, 7]. The two-component systems (TCS) consist of a light-sensitive sensor kinase (SK) that regulates its response regulator (RR), in these cases a transcription factor (TF), by means of phosphorylation. Potential limitations of TCS are their relatively large size and complexity; the reliance of RR activity on specific phosphorylation by the SK exposes the system to the risk of cross-talk with other TCS, and expression levels of the SK and the RR may have to be individually tuned [7]. Recently, light-induced transcription factors have been engineered [8–10], which do not require membrane localization. They rely on light-induced dimerization to bind and positively regulate their target promoters. The simplest type of such optogenetic dimer activators is the one-component systems based on protein homodimers, which have recently been engineered for mammalian cells by fusing activation domains to the light-sensor proteins VVD [8] and EL222 [9]. Because of the relative simplicity and small size of one-component optogenetic systems, they are an attractive option for light-regulated systems that could be used in several different host strains or cell types. However, as activation of gene expression requires interaction with host-specific TFs and/or RNA polymerases (RNAP), gene activation systems are limited in the number of hosts they can function in. Repression of gene expression, on the other hand, whose simplest mode of action is exclusion of RNAP from the promoter by steric hindrance, is because of its simplicity likely a widespread if not universal mechanism. Up until lately, potential one-component optogenetic repression systems were either not implemented *in vivo* [11] or repressed gene expression *in vivo* with limited efficiency [12].

Recently, however, several one-component optogenetic repression systems that function *in vivo*, incorporating the light sensors VVD, RsLOV, and EL222, respectively, have been presented [13–16]. The system with the highest promoter repression ratio was based on VVD and the *Escherichia coli* (*E. coli*) LexA repressor. It was found to regulate the mRNA levels of target genes up to 10,000-fold, illustrating the potential of one-component optogenetic repression systems [13]. The EL222-based optogenetic repression systems, in comparison, displayed repression ratios of about 4- to 53-fold [15, 16]. Nonetheless, the EL222 TF possesses several characteristics that could make it a highly efficient one-component optogenetic repressor. It is a small protein of 222 amino acids from the bacterium *Erythrobacter litoralis* HTCC 2594 and consists of a blue light-sensitive light-oxygen-voltage (LOV) domain and a helix-turn-helix DNA-binding domain [17]. Upon stimulation by blue light, EL222 forms a dimer and binds its DNA target site [18], for which 12 bp consensus sequence was found to be RGNCYWWRGNCY (where R=A or G, N any nucleotide, Y=C or T, and W=A or T), with submicromolar affinity *in vitro* [19]. Because of the tight and light-regulated DNA target site binding of EL222, we hypothesized that a promoter carrying the EL222 target site could be rendered light-regulated. For this, we considered BBa_J23119 (J23119) (iGEM Registry of Standard Biological Parts), one of the most often used constitutive promoters in synthetic biology due to its strength and clean transcription start. Our synthetic promoter could be used instead of the J23119 promoter in dozens of different gene circuits, empowering them with a light input. We also discuss the characteristics of the herein presented system and compare with the two recently published one-component optogenetic repression systems [13, 15]. Finally, we suggest ways to improve EL222-based systems, as well as systems built on other one-component optogenetic repressors, for use as transcriptional tools.

## 2. Materials and Methods

### 2.1. Strains and Culture Conditions

*E. coli* strain DH5*α*Z1 (Expressys), which carries two *lacI^q^* genes at the *attB* locus, was used for cloning and characterization. Cultures were grown at 37°C in LB medium, or on LB-agar plates for cloning purposes, and in LB or supplemented M9 medium (M9S) (100 *μ*M CaCl_2_, 2 mM MgSO_4_, 1X M9 salts, 0.8% v/v glycerol, 0.2% w/v casamino acids and 1 mM thiamine) at 37°C for characterization purposes. 1X M9 salts contain 47.8 mM Na_2_HPO_4_, 22 mM KH_2_PO_4_, 8.6 mM NaCl, and 18.6 mM NH_4_Cl. The medium was supplemented with 25 *μ*g/ml kanamycin for pPMQAK1 and with 25 *μ*g/ml chloramphenicol for pSB1AC3 or both antibiotics when the plasmids were used in combination.

### 2.2. Construction of the P*_EL_* Reporter and EL222 Expression Constructs

First, a preliminary construct consisting of fused P*_EL_* reporter and EL222 expression cassettes was made. The P*_EL_* reporter cassette was made with PCR using PrimeSTAR HS DNA polymerase (Takara) (PrimeSTAR) with primers P(EL119CP)-sfGFPaav-f1 and aav-B1006-r1 (oligonucleotide sequences are available in Table S1) and plasmid DNA containing the sfGFP coding sequence [20] with an LAA protein degradation tag [21] as template. The EL222 coding DNA sequence from *Erythrobacter litoralis* HTCC 2594 corresponding to amino acids 14-222 was codon-optimized and synthesized (Integrated DNA Technologies) together with an RBS and a transcriptional terminator. The synthetic EL222 sequence was used as template in a PrimeSTAR reaction using primers PLlacSyn-EL222-f1 and rnpBT1-r1.The resulting product was cloned with the P*_EL_* reporter cassette product into pPMQAK1 [22] using BioBrick 3A assembly [23]. This preliminary, combined construct was then used as template in subsequent PCRs to produce the final P*_EL_* reporter and EL222 expression constructs. The P*_EL_* reporter construct was amplified with Phusion HS II DNA polymerase (Life Technologies) (Phusion) and with primers P(EL119CP)-sfGFPaav-f1 and aav-B1006-r1. The EL222 expression construct was produced in two Phusion PCRs and in two versions using either primer P1OcBCD2EL-f1 (without flag-tag) or primer P1OcBCD2FlgEL-f1 (with flag-tag) and reverse primer rnpBT1-r1. In the second PCR, the final two versions of the EL222 expression construct were produced using the first products as templates and primers X-P1OcBCD2-f2 and rnpBT1-r1. The P*_EL_* reporter construct was cloned into the BioBrick site of pPMQAK1, and the two versions of the EL222 expression construct were cloned into the BioBrick sites of pSB1AC3 [23] and pPMQAK1. Finally, the combined P*_EL_* reporter and EL222 expression construct was constructed by 3A assembly of the individual P*_EL_* reporter and EL222 expression constructs into pPMQAK1 (construct sequences are available in Supplementary Materials: sequences of parts and constructs).

### 2.3. Characterization of EL222 Expression and Fluorescence

Three biological replicate cultures of the EL222 expression construct on pSB1AC3 with or without an N-terminal flag-tag, and of the empty pSB1AC3 vector (empty vector), were grown in LB overnight until stationary phase. Experimental cultures were started by diluting cultures 1 : 200 in LB and inoculating 200 *μ*l into a black 96-well plate with clear flat bottom (BD Falcon) with or without 2 mM IPTG induction. The plate was incubated shaking at level 6 (approximately 850 rpm) on a Delfia plate shaker (Wallac) (shaker) in darkness, and measurements were taken using a Chameleon V Microplate Plate Reader (Hidex). Culture absorbance at 595 nm (A595) and EL222 fluorescence, measured using 485 nm emission and 535 nm excitation filters, were recorded at 0, 1.18, 2.52, 3.48, and 4.62 hours after the start of the experiment. After subtracting medium background, the fluorescence per cell density was calculated by normalizing fluorescence with A595. Specific EL222 fluorescence per cell density values for the different strains was calculated by subtracting the fluorescence per cell density of the empty vector strain. Averages and standard deviations from the mean were calculated for three biological replicates (data is available in Data file S1).

For characterization of the EL222 fluorescence emission spectrum, LB cultures of the empty vector and the Flag-EL222 expression constructs were grown overnight and diluted 1 : 200 in LB in the morning. The cultures were grown for 6 hours and then induced with 0.2 mM IPTG for two hours. The cells were collected by centrifugation at 4°C, resuspended in lysis buffer (100 mM NaCl, 5 mM EDTA, 30 *μ*l/ml protease arrest (Calbiochem), and 50 mM Tris-HCl pH 8.0), and lysed by sonication using a Vibra-Cell instrument (Sonics) on ice at 6 W pulses of 2 s for 1 min, which was repeated once with 1 min rest in between. The lysate was cleared by centrifugation, extracted, and stored at -20°C. For characterization of the EL222 fluorescence emission spectrum, the empty vector and the Flag-EL222 lysates were diluted with lysis buffer containing 10 *μ*l/ml protease arrest to the same protein concentration (as estimated by absorption at 280 nm) and to an absorption at 485 nm<0.1. Fluorescence emission measurements were taken with a Fluorolog-3 spectrofluorometer (Horiba Jobin Yvon) using a 450 nm filter for excitation and collecting emission from 460 to 700 nm. The fluorescence spectrum of EL222 was calculated by subtracting the empty vector lysate fluorescence spectrum from the Flag-EL222 lysate spectrum and normalizing all values to the maximum value (data is available in Data file S2).

### 2.4. Detection of EL222 Expression and Repression of P*_EL_*

Three cultures each of the P*_EL_* reporter construct on pPMQAK1 and of cultures containing both the P*_EL_* reporter construct on pPMQAK1 and the EL222 expression construct on pSB1AC3 and one culture of the empty pPMQAK1 vector were grown overnight until stationary phase. The cultures were diluted 1 : 200 into 3 ml LB in growth tubes in the morning and grown shaking for 6.5 hours in strong white light (spectrum is available in Figure S1 and the data in Data file S3) of ca 150-200 *μ*mol photons m^-2^ s^-1^ to activate EL222 dimerization. A595 was measured, and cells were harvested by centrifugation at 4°C and stored at -20°C. The cell pellets were resuspended in sodium dodecyl sulfate (SDS) polyacrylamide gel electrophoresis (PAGE) sample buffer with 2-mercaptoethanol to a cell density corresponding to the same A595 value and denatured at 95°C for 6 minutes. The samples were identically loaded on three any-kD PAGE gels (Bio-Rad) that were run at 200 V for 45 minutes, after which one gel was Coomassie-stained using PageBlue (Life Technologies) and two gels were blotted to PVDF membranes using Trans-Blot Turbo packs (Bio-Rad). Each blotted membrane was probed for either GFP or Flag-EL222 using polyclonal GFP antibodies from rabbit (G1544, Sigma-Aldrich, diluted 1 : 2000) in conjunction with Immun-Star HRP-conjugated anti-rabbit secondary antibodies from goat (1705046, Bio-Rad, diluted 1 : 5000),or monoclonal flag-tag antibodies from mouse (F3165, Sigma-Aldrich, diluted 1 : 3500) in conjunction with HRP-conjugate anti-mouse secondary antibodies from rabbit (ab6728, Abcam, diluted 1 : 10000), per the manufacturer’s instructions. The western immunoblots were developed using the Immun-Star HRP substrate kit (Bio-Rad) and imaged using a ChemiDoc system (Bio-Rad).

### 2.5. Time-Course Characterization of Light-Regulated EL222 Repression of P*_EL_*

Six replicate cultures of each of the empty pPMQAK1 vector, the EL222 expression construct on pPMQAK1, the P*_EL_* reporter construct, and the combined P*_EL_* reporter and EL222 expression construct were grown in M9S overnight until stationary phase. In the morning, the cultures were diluted 1 : 100 in 200 *μ*l M9S in a black 96-well plate with clear flat bottom covered by a black lid that blocks all light (darkness plate) and in a translucent 96-well tissue culture plate (Sarstedt) covered with a translucent lid to allow for light penetration (light plate). The plate cultures were grown shaking at level 6 (approximately 850 rpm) on the plate shaker in white light of ca 80 *μ*mol photons m^-2^ s^-1^. A595 and fluorescence, using 485 nm excitation and 535 nm emission filters, were measured using the plate reader every second hour up until 10 hours. Medium background was subtracted, and fluorescence per cell density was calculated by normalizing fluorescence with A595. Means and sample standard deviations from the mean were calculated for six biological replicates, except for one combined P*_EL_* reporter and EL222 expression construct replicate on the darkness plate, which was excluded due to plate loading error. Specific GFP fluorescence per cell density values for the P*_EL_* reporter construct was calculated by subtracting the empty vector background fluorescence, and new standard deviations for the differences were calculated. Specific GFP fluorescence per cell density values for the combined P*_EL_* reporter and EL222 expression construct was calculated by subtracting the EL222 expression construct on pPMQAK1 background fluorescence, and new standard deviations for the differences were calculated. To account for potential bleaching effects, the specific GFP fluorescence of the combined construct was normalized with the specific GFP fluorescence of the P*_EL_* reporter construct, in darkness and light, respectively, and the standard deviations of the ratios were estimated assuming independent samples. Finally, light-regulated repression ratios were calculated by dividing the normalized values in darkness with the normalized values in light (data and calculations are available in Data file S4).

### 2.6. EL222 Repression Reversibility Assay

Duplicate LB cultures of the combined P*_EL_* reporter and EL222 expression construct and of the P*_EL_* reporter construct were grown overnight until stationary phase. In the morning, the cultures were diluted 1 : 200 into 10 ml LB in 50 ml Falcon tubes with loose lids. The Falcon tube cultures were grown shaking for two hours in darkness and then exposed to three cycles of one hour of strong white light of ca 150-200 *μ*mol photons m^-2^ s^-1^ and one hour of darkness. Samples were extracted in the end of each phase for A595 measurements, cells were collected by centrifugation at 4°C, and cell pellets were stored on ice. After the last cycle, the cell pellets were resuspended in SDS-PAGE sample buffer with 2-mercaptoethanol and denatured at 95°C for 6 minutes. The time-series samples of the combined P*_EL_* reporter and EL222 expression construct and of the P*_EL_* reporter construct were loaded on two different any-kD PAGE gels in volumes corresponding to the same number of cells as approximated from the A595 values. The gels were run at 200 V for 45 minutes, blotted to PVDF membranes using Trans-Blot Turbo packs, and probed for GFP using the same protocol as mentioned in Detection of EL222 Expression and Repression of P*_EL_*. After the Western immunoblots, the membranes were stained for protein using PageBlue per the manufacturer’s instructions and scanned.

## 3. Results

We exploited the capability of the transcription factor EL222 to undergo light-stimulated dimerization and subsequently strongly bind its DNA target, to engineer a repressible optogenetic one-component system. Using the principle of transcriptional repression via steric hindrance [24], we designed a promoter where the binding of EL222 competes directly with the binding of RNAP. When EL222 is present in the cell, the transcriptional output from such a promoter would depend on whether EL222 is bound or not. As the capacity of EL222 to form a dimer and bind the promoter is controlled by light, we hypothesized that such a transcriptional system would be light-regulated.

**Figure 1:**
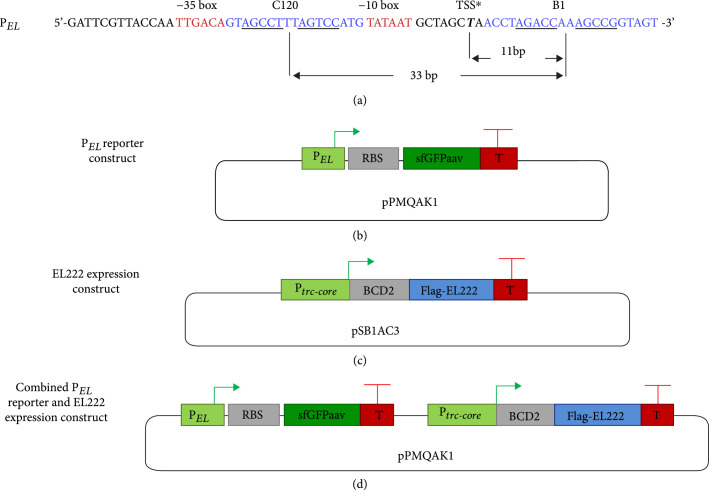
Design schemes. (a) The 5${}^{\prime }$ to 3${}^{\prime }$ DNA sequence of the synthetic P*_EL_* promoter and its elements. The promoter’s -35 and -10 boxes are marked in red letters, and a putative transcription start site (TSS∗) is marked in bold italic. The two EL222 operators C120 and B1 are marked in blue letters, and the 5-base pair (bp) binding motifs are underscored. Arrows indicate the distances in bp between centers of operators or the TSS∗ and the center of the B1 operator. (b) Design of the P*_EL_* reporter construct consisting of the P*_EL_* promoter, a ribosome binding site (RBS), superfolder GFP with an AAV degradation tag (sfGFPaav), and finally a transcriptional terminator (T). The P*_EL_* reporter construct is carried on the low-copy number plasmid pPMQAK1. (c) Design of the EL222 expression construct consisting of the P*trc-core* promoter, a bicistronic translation initiation element (BCD2), EL222 without or with an N-terminal flag-tag (Flag-EL222), and a terminator. The EL222 expression construct is carried on the high-copy number plasmid pSB1AC3 or on pPMQAK1 (not shown). (d) Design of the combined P*_EL_* reporter and EL222 expression construct consisting of the respective constructs in (b) and (c) separated by a short scar sequence. The combined construct is carried on the low-copy pPMQAK1 plasmid.

We designed a synthetic promoter, P*_EL_*, to be repressible by EL222. For this, we made use of the synthetic promoter J23119 that previously has been utilized as a scaffold to construct repressible promoters [25]. To facilitate EL222 binding, two EL222 operators were inserted into the core of and downstream of J23119, respectively (Figure 1(a)). A truncated version of the C120 operator 5${}^{\prime }$-TAGGTAGCCTTTAGTCCATG-3${}^{\prime }$, a maximum affinity EL222-binding consensus sequence [19], was inserted between the -35 and -10 elements of J23119 (EL222-binding site motifs underscored). In this position, EL222 will directly compete with RNAP for binding the promoter. For selecting a second operator, we sought to minimize the sequence homology with C120, as a common mechanism for genetic instability is recombination between repeated or homologous sequences [26]. Therefore, we selected the peak B EL222-binding sequence 5${}^{\prime }$-ACCTAGACCAAAGCAGGTAGT-3${}^{\prime }$ [19], which displays limited homology to C120 (alignments are available in Figure S2). However, the second 5 bp EL222-binding site does not conform to the consensus 5 bp “RGNCY” binding site motif [19]. To potentially increase the affinity of EL222 to the peak B sequence by partially restoring the consensus binding motif, we substituted the fourth “A” of the second binding site to “C,” thereby creating operator B1. We inserted the B1 operator 1 bp downstream of the putative transcription start site (TSS∗) of J23119 (Figure 1(a)). This centers B1 11.5 bp from the TSS∗ or approximately one DNA helix turn away from the TSS∗, assuming the previously reported average value of ~11 bp per turn of *E. coli in vivo* chromatin DNA [27, 28]. This was done in analogy with the well-repressed *E. coli lacZYA* promoter, where the primary *lac* operator is centered one turn away from the TSS. Additionally, the centers of the C120 and B1 operators are separated by 33 bp, or an estimated three helix turns, to facilitate any potentially stabilizing interactions between two EL222 dimers bound on the same face of the DNA (Figure 1(a)). To enable the time-resolved study of EL222-regulated P*_EL_* activity, a promoter reporter cassette based on a superfolder green fluorescent protein (GFP) [20], tagged with an *ssrA*-based protein degradation tag [21] to minimize cell growth artifacts, was constructed and cloned on the low-copy pPMQAK1 vector [22] (Figure 1(b)). For characterization of EL222 expression and fluorescence, untagged and flag-tagged versions of EL222, codon optimized for *E. coli* and *Synechocystis* PCC 6803, were designed. P*trc-core*, a leaky LacI-regulated promoter that ends with its TSS and can be induced to higher expression levels [29], and BCD2, a translation initiation element that has been previously shown to partially decouple translation initiation from interactions with the beginning of the coding sequence [30], were used to drive EL222 expression. The EL222 expression constructs were cloned on the high-copy number plasmid pSB1AC3 [23] and on pPMQAK1 (Figure 1(c)). Finally, the P*_EL_* reporter and EL222 expression constructs were cloned in combination on pPMQAK1 to enable coexpression of EL222 from the same plasmid as the promoter reporter construct (Figure 1(d)).

**Figure 2:**
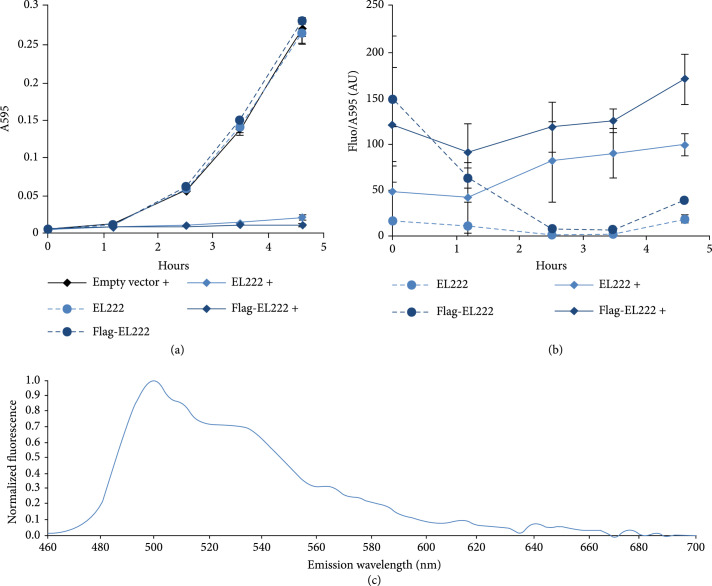
Characterization of growth and fluorescence of EL222-expressing *Escherichia coli* cell cultures. (a) Growth curves measured in absorbance at 595 nm (A595) of the DH5*α*Z1 strain (*lacI^q^*) containing empty pSB1AC3 (empty vector) or pSB1AC3 carrying the EL222 expression construct without (EL222) or with an N-terminal flag-tag (Flag-EL222). Cultures where 2 mM isopropyl *β*-D-1-thiogalactopyranoside (IPTG) was added to induce EL222 expression above the leakage levels of the P*trc-core* promoter are marked with “+.” (b) EL222 fluorescence per cell density and time measured as fluorescence normalized with A595 (Fluo/A595) in the strains described in (a). Error bars in both (a) and (b) denote standard deviation of the mean, n=3, AU (arbitrary units). (c) Normalized fluorescence emission spectrum of EL222 in a whole cell lysate of the Flag-EL222 expressing strain described in (a), excited with 450 nm light.

To study the effects of EL222 expression on cell growth in the *E. coli lacI^q^* strain DH5*α*Z1 (Expressys), we compared the growth of empty pSB1AC3 control cultures with that of untagged or flag-tagged EL222 pSB1AC3-carried expression construct cultures (Figure 2(a)). The noninduced leakage expression of EL222 from the P*trc-core* promoter was not enough to affect growth as compared with the empty vector control cultures. However, induction of EL222 expression using 2 mM isopropyl *β*-D-1-thiogalactopyranoside (IPTG) leads to severe growth retardation, implying that high levels of EL222 expression are detrimental to the cells. Further, as blue light-absorbing LOV-domain proteins are often fluorescent enough to be used as reporters themselves [31], and since such fluorescence could affect the use of other fluorescent protein reporters, we investigated the fluorescence levels of EL222-expressing cultures using the same filter settings as used for subsequent GFP measurements (Figure 2(b)). Here, differences in fluorescence should be evaluated with caution, as cultures were grown in LB which leads to increased cell growth-dependent noise. Still, as could be predicted from the growth curves, the induced cultures clearly express the most EL222, as judged by the higher levels of fluorescence normalized to cell density during exponential growth (around 2.5 hours) compared with the noninduced cultures. Further, the flag-tagged EL222 constructs consistently displayed stronger normalized fluorescence than the untagged constructs, implying that the flag-tag improves expression of EL222. Hence, further experiments involved only the use of the flag-tagged EL222 expression construct, which also facilitates detection of EL222 expression with flag-tag antibodies and Western immunoblotting. Lastly, to determine the potential fluorescence interference from EL222 to the use of fluorescent reporters, we measured the fluorescence emission spectrum of Flag-EL222 in a total protein extract from an EL222-expressing DH5*α*Z1 culture (Figure 2(c)). For excitation, we used 450 nm light, which was established as the maximum absorption peak by a previous study [17]. While the main fluorescence emission peak is at 500 nm, there are secondary peaks and significant fluorescence all the way into the green and yellow parts of the visible light spectrum, with the fluorescence leveling out from ca 600 nm (fluorescence emission data is available in Data file S2). This means that the accurate characterization of reporter fluorescence in the presence of EL222 requires subtraction of interfering fluorescence from EL222, as was done in this study, or the use of red to far-red reporters.

**Figure 3:**
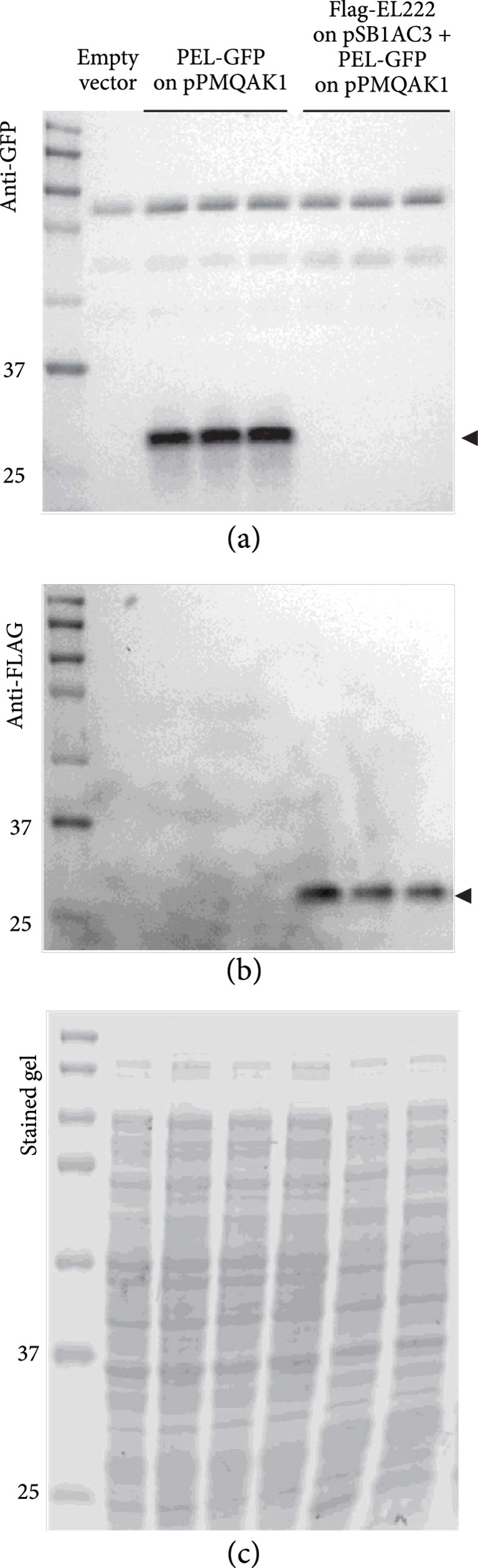
Western immunoblots/SDS-PAGE demonstrating the expression of Flag-EL222 and its capability to repress expression of GFP from the P*_EL_* promoter *in vivo*. Each of the lanes in the three panels correspond to identical protein samples from engineered *Escherichia coli* DH5*α*Z1 cells grown under constant white light. (a) Western immunoblot against GFP. (b) Western immunoblot against the Flag-tag on EL222. (c) Coomassie-stained SDS-PAGE gel. “kDa”: kilodaltons; “L”: ladder/protein size marker; “Empty vector” corresponds to pPMQAK1 only, “P*_EL_*-GFP on pPMQAK1” to the P*_EL_* reporter construct, and “Flag-EL222 on pSB1AC3” to the EL222 expression construct with an N-terminal Flag-tag. “+” indicates simultaneous presence of both plasmids. The strains with the P*_EL_* reporter construct alone and in combination with the EL222 expression construct were grown in biological triplicates. The expected size of GFP with an AAV tag is ca 29 kDa and of Flag-EL222 ca 24 kDa.

Next, we used Western immunoblotting to investigate the capability of Flag-EL222 expressed from pSB1AC3 to repress expression from the P*_EL_*-GFP reporter construct on pPMQAK1 (Figure 3). We probed blotted protein extracts from cultures of the P*_EL_*-GFP reporter construct alone or cotransformed with the Flag-EL222 expression construct, with both GFP and Flag-tag antibodies. In addition, to activate the dimerization capability of EL222, the cultures were grown in intense white light (see Figure S1 for spectrum; data is available in Data file S3). During gel running, there was a slight distortion of the lowest part of the SDS-PAGE gel used for staining (Figure 3(c)). However, the gels used for blotting were not distorted and it is apparent from the stained gel that the wells were equally loaded. Interestingly, GFP expression from the P*_EL_*-GFP reporter construct on the low-copy pPMQAK1 vector was completely repressed (Figure 3(a)) by the leakage levels of Flag-EL222 expressed by P*trc-core* from the high-copy pSB1AC3 vector (Figure 3(b)). However, while this confirms that EL222 can act as a repressor of the synthetic P*_EL_* promoter, it does not confirm that repression is light-regulated.

**Figure 4:**
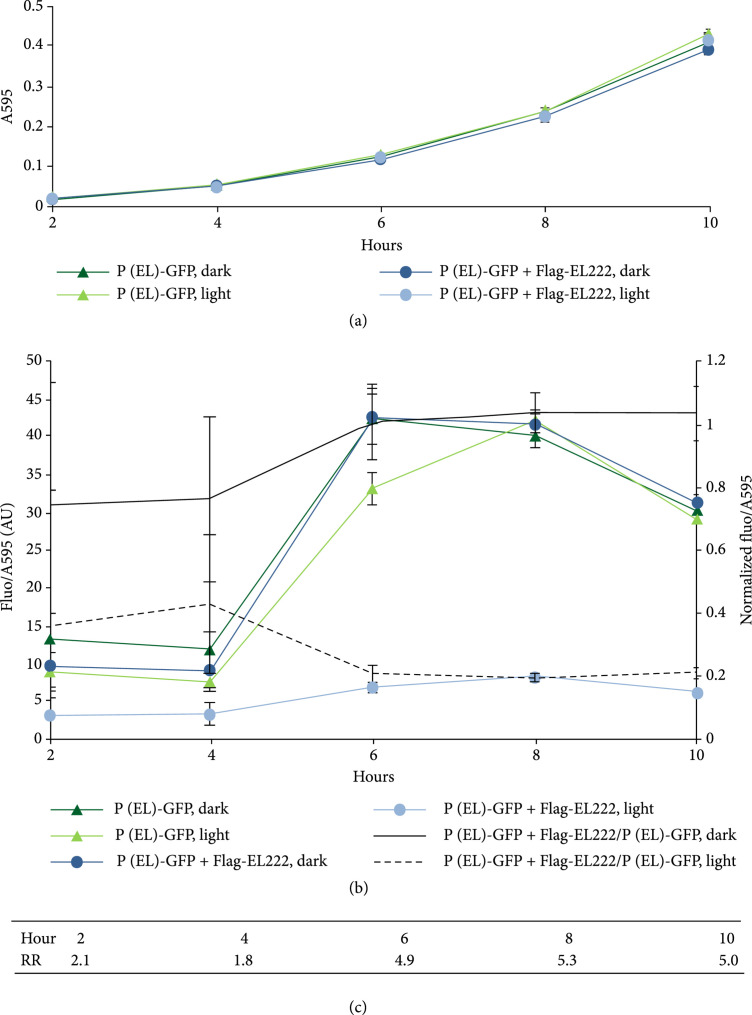
Time-course characterization of light-regulated and EL222-mediated P*_EL_* repression. The engineered *Escherichia coli* DH5*α*Z1 cell cultures grew in darkness (dark) or under constant white light (light). (a) Growth curves measured in absorbance at 595 nm (A595) of the P*_EL_* reporter construct only (“P(EL)-GFP”) or cloned together with the Flag-EL222 expression construct (“$\text{P}\left(\text{EL}\right)-\text{GFP}+\text{Flag}-\text{EL}222$”), all carried on the low-copy pPMQAK1. (b) GFP fluorescence per cell density time development measured as fluorescence normalized with A595 (Fluo/A595) in the strains described in (a). Error bars in both (a) and (b) denote the standard deviation of the mean, n=6, AU (arbitrary units). The markerless black lines indicate the ratio of the fluorescence per cell density of the combined P*_EL_* reporter and EL222 expression construct normalized with the fluorescence per cell density of the P*_EL_* reporter construct, in darkness (continuous line) or light (dashed line). Error bars denote estimated ratio standard deviations. (c) Light-regulated repression ratios (RR) computed from the normalized fluorescence per cell density values in darkness divided by the corresponding values in light.

To test if Flag-EL222-mediated repression of P*_EL_*-GFP is light-regulated, we performed a time-course characterization study comparing the effects of light and darkness, when all constructs are carried on the low-copy pPMQAK1 vector. First, we investigated if cultures bearing only the reporter construct, or bearing the combined reporter and Flag-EL222 expression construct, grew differently in light and darkness. However, no difference in growth could be detected between the constructs or between conditions of light and darkness (Figure 4(a)). As a control for GFP bleaching, we then compared GFP fluorescence normalized to cell density from the P*_EL_*-GFP reporter construct in light and darkness. A decrease in fluorescence/cell density in light as compared with darkness could be observed at the earlier time points (Figure 4(b)). This implies that there is some bleaching of GFP at lower cell densities. As this difference between light and darkness disappears for later time points, we hypothesize that cell shading due to higher cell densities can then protect the GFP fluorophore. Further, to specifically study GFP fluorescence in the combined P*_EL_* reporter and Flag-EL222 expression cultures, EL222 background fluorescence was subtracted. As the fluorescence of EL222 will depend on whether it is exposed to light or not, we subtracted the EL222 background fluorescence of cultures grown under the same conditions of light and darkness as the respective combined test construct cultures. The combined P*_EL_* reporter and Flag-EL222 expression construct, interestingly, displays a clear fluorescence/cell density dependence on light, implying that the P*_EL_* promoter is indeed light-regulated. To compensate for GFP bleaching, we normalized the fluorescence/cell density values of the combined construct with the values of the reporter-only construct in darkness and light, respectively (Figure 4(b)). If Flag-EL222 has no effect on expression from P*_EL_*, which should be the case for darkness given that EL222 is light-regulated, the normalized value should approach one. Yet, at two and four hours after start of the experiment, the normalized fluorescence/cell density values in darkness are around 0.75. However, the fluorescence levels at these early time points are low, allowing noise to have a large impact on the ratio. At later time points from six hours and on, though, this ratio stabilizes at one, as expected for an inactive EL222. Conversely, in light, the normalized fluorescence/cell density values are consistently lower than 1, stabilizing at ca 0.2 from six hours and on. Although there is some leakage from the Flag-EL222-repressed P*_EL_* promoter, the dependence of repression on light is clear, with a light-regulated repression ratio stabilizing at ~5 (Figure 4(c)).

**Figure 5:**
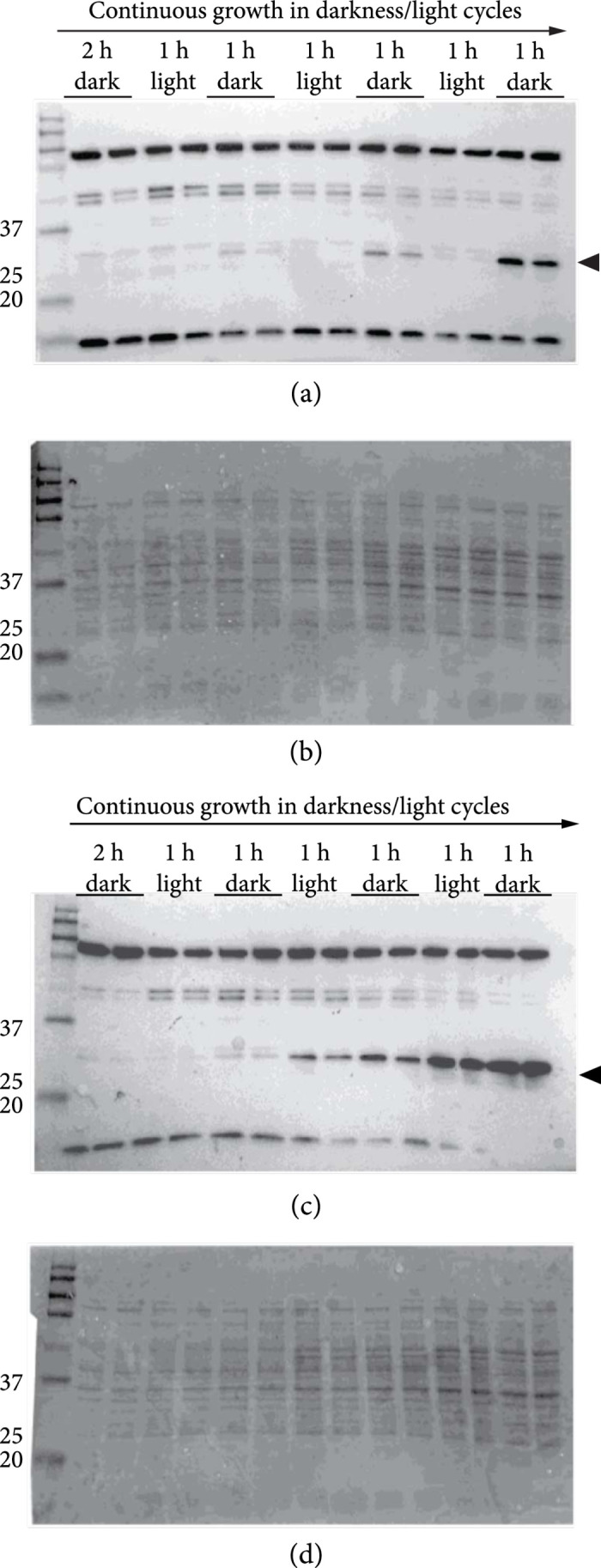
Anti-GFP Western immunoblots and stained membranes demonstrating the reversibility of light-regulated EL222-mediated P*_EL_* repression in engineered *Escherichia coli* cells. Duplicate DH5*α*Z1 cultures of the P*_EL_* reporter construct (“P*_EL_*-GFP”) and the combined P*_EL_* reporter and Flag-EL222 expression construct (“${\text{P}}_{EL}-\text{GFP}+\text{Flag}-\text{EL}222$”) were grown for two hours in darkness and then subjected to three growth cycles consisting of one hour in white light and one hour in darkness. All constructs were carried on the low-copy pPMQAK1 plasmid. Samples were taken in the end of each phase, which is accordingly annotated (top, dark-phase samples underscored). After probing for GFP, the membranes were stained for total protein. (a) Immunoblot against GFP from ${\text{P}}_{EL}-\text{GFP}+\text{Flag}-\text{EL}222$ cultures. (b) Stained membrane from (a). (c) Immunoblot against GFP from P*_EL_*-GFP cultures. (d) Stained membrane from (c). Arrowheads denote the position of GFP band; numbers represent size in kilodaltons of the protein size marker. The expected size of GFP, with an AAV-degradation tag, is ca 29 kDa.

Finally, we investigated the reversibility of light-regulated EL222 repression of P*_EL_* using a time-course study with cycling conditions of darkness and light. Because of the protein degradation tag on the otherwise stable GFP and because of dilution due to cell division, the levels of GFP per cell are expected to fall upon repression of P*_EL_*-GFP expression. Cultures containing the P*_EL_* reporter only, or the combined P*_EL_* reporter and Flag-EL222 expression construct, were subjected to cycles of darkness and light. To measure the presence of GFP while circumventing GFP bleaching-related problems, culture samples taken in the end of each phase were denatured and used for Western immunoblots against GFP (Figure 5). We found that the GFP levels of the combined construct decreased periodically with the presence of light, in darkness/light cycles as brief as one hour. The P*_EL_*-GFP reporter only cultures, on the other hand, did not display light-dependent cyclically varying levels of GFP.

## 4. Discussion

We have presented a novel synthetic promoter that could enable synthetic gene circuits to use light as an inducer. As our promoter is based on the popular synthetic promoter J23119, it may be used as its replacement, facilitating light-regulated gene expression tuning. Our synthetic promoter P*_EL_* in combination with its cognate transcription factor EL222 has a similar or slightly improved repression ratio as compared with other synthetic light-repressible promoters reported previously [12, 15]. However, a later study (the LightOff system) has proposed an alternative transcription factor consisting of a fusion of the light sensor protein VVD to the endogenous repressor LexA from *E. coli*. Together with a LexA-regulated promoter, it repressed the mRNA levels up to 10,000-fold [13]. Unfortunately, the use of an endogenous DNA-binding domain risks producing cross-talk. While it was shown that the native LexA did not interfere appreciably with a LightOff promoter reengineered to bind the mutated LexA domain, it is possible that the native and mutated LexA domains of the LightOff system interact with native LexA-binding partners in the cell. The risk for unpredictable effects stemming from interactions with host cell machinery is potentially lower for exogenous transcription factors, like in the present study EL222 in *E. coli*, as they have not evolved to function in the host chassis. In a recent update of the LightOff system (the eLightOn system), the LexA DNA-binding domain was instead fused to the *Rhodobacter sphaeroides* blue light sensor RsLOV [14]. The resulting transcription factor forms a dark-state dimer capable of binding its operator, which disassociates when exposed to blue light. This was used to engineer a darkness-repressed promoter that could be induced 500-fold upon light exposure. While this system is also poised to be a useful tool for light-regulated transcription, eLightOn suffers the same potential LexA-related risk as LightOff. Nevertheless, the LightOff and eLightOn systems illustrate the great potential of one-component optogenetic repression systems. They also suggest that the P*_EL_*-EL222 system herein presented and likewise the PBLrep-v1 system presented by Jayaraman et al. [15] and the blue light repression tool (BLRT) presented by Ding et al. [16] have margins of improvement. Indeed, we expect that all these EL222 systems can be further developed by optimizing several parameters important for the characteristics of any optogenetic promoter-TF couple. Considering the regulated promoters per se, P*_EL_* and PBLrep-v1 could likely be further optimized to provide stronger expression and higher-fold regulation. After further optimization, the P*_EL_*, which contains two different EL222 operators as opposed to PBLrep-v1 and BLRT which contain only one, could potentially be repressed more strongly, as the binding and activity of RNAP would be hindered in two positions instead of one. Also, P*_EL_* was designed to enable the possibility of stabilizing interactions between two EL222 dimers occupying both operators simultaneously. To optimize P*_EL_*, a combinatorial approach investigating the position and combination of different EL222 operator sequences and core promoter elements could be performed as inspired by previous studies [16, 32]. Further, PBLrep-v1, BLRT, and an optimally refactored P*_EL_* could be fine-tuned through a directed evolution approach [33]. Alternatively, instead of evolving the promoter sequence or the EL222 operators themselves, the DNA-binding specificity of EL222 could be engineered using phagemid-based directed evolution [34]. Another critical optimization parameter of a repressed promoter system is the relative ratio of TF molecules versus the number of target operators on the promoter. Ideally, the number of TF molecules should be high enough to maximize repression of the promoter while allowing for the maximal release of repression upon induction. In the case of EL222, the concentration of EL222 molecules should be sufficiently high as to provide light-regulated dimerization and maximize binding to and repression of the promoter. But also, care must be taken to maintain the concentration of EL222 below an unknown threshold above which could commence dimerization and operator binding in darkness. For an EL222-regulated promoter, this would lead to lower maximal expression levels in darkness, which in turn leads to lower light-regulated repression ratios. For the combined P*_EL_* reporter and Flag-EL222 expression construct used in this study, EL222 expression levels were not high enough to completely abolish P*_EL_* expression in light (Figures 4 and 5). Also, for the PBLrep-v1 system, reporter gene expression could not be completely repressed by EL222 [15]. These results indicate the need for higher concentrations of EL222 for improved repression. Our investigation of cell growth during different EL222 expression levels implies that high levels of EL222 expression can be detrimental but that non-growth-retarding levels can be achieved without induction using the present EL222 construct carried on a high-copy plasmid (Figure 2(a)). Further, this level of EL222 expression is enough to completely repress P*_EL_* expression from a low-copy vector (Figure 3). Hence, it is likely that raising the EL222 expression levels from that of the combined P*_EL_* reporter and Flag-EL222 expression construct on the low-copy pPMQAK1 plasmid will improve repression. An effective strategy to optimize EL222 levels would be to insert the P*_EL_* reporter on a single-copy vector or the chromosome and then adjust EL222 expression as high as possible without causing dark state dimerization or growth retardation. In addition to the EL222 expression levels, the EL222 protein itself could be modified to provide improved repression characteristics or a change in repression kinetics. For instance, while the light-activated EL222 completely decays to the dark state in one to two minutes [17, 35], enabling the adoption of our system for rapid time-resolved studies, it is possible to modify the light-regulated kinetics by engineering or evolving the LOV domain [36, 37]. Further, other aspects important for EL222 activity such as light intensity and quality should be optimized, as was recently done comprehensively for the PBLrep-v1 system [15] and for the BLRT [16]. Furthermore, EL222 in combination with synthetic promoters carrying EL222 operators could, with the abovementioned optimization strategy, be introduced into other hosts of relevance to synthetic biology. There, they could provide orthogonal one-component optogenetic repression alternatives. Additionally, for any host chassis, it would be helpful to benchmark and even normalize light-regulated promoter expression to that of commonly used standard reference promoters, as was done previously in *E. coli* [38]. To conclude, an optimized EL222-regulated gene repression system could become a powerful yet simple optogenetic tool to empower synthetic gene circuits to be regulated by light instead of using a two-component system localized in the plasma membrane.

## Data Availability

DNA sequence data is supplied within the paper and in the supplementary materials. Raw data is supplied as supplementary data files.

## Supplementary Materials

Supplementary Materials Figure S1: spectrum of white light normalized to maximum photon counts. The underlying data is available in Data file S3. Figure S2: alignment of the C120 operator with the peak band B1 operators. The EL222-binding site motifs are underscored. Table S1: DNA oligonucleotide sequences. Data file S1: plate reader data from the characterization of EL222 expression and fluorescence. Data file S2: the EL222 fluorescence emission data. Data file S3: data of white light used for EL222 repression assays. Data file S4: plate reader data and data analysis from the time-course characterization of light-regulated EL222-mediated P*_EL_* repression.
