# Supplementary material for: Engineering of a Promoter Repressed by a Light-Regulated Transcription Factor in Escherichia coli
Source: Biodes Res. 2021 Sep 28;2021:9857418. doi: 10.34133/2021/9857418 (PMC10521638; doi:10.34133/2021/9857418)
Supplement: Supplementary Materials — Figure S1: spectrum of white light normalized to maximum photon counts. The underlying data is available in Data file S3. Figure S2: alignment of the C120 operator with the peak band B1 operators. The EL222-binding site motifs are underscored. Table S1: DNA oligonucleotide sequences. Data file S1: plate reader data from the characterization of EL222 expression and fluorescence. Data file S2: the EL222 fluorescence emission data. Data file S3: data of white light used for EL222 repression assays. Data file S4: plate reader data and data analysis from the time-course characterization of light-regulated EL222-mediated PEL repression. [file 9857418.f1.zip › Supplementary Material.docx]

Supplementary Materials

**Supplementary sequences of parts and constructs**

P*EL* reporter construct

P*EL*(EL222 binding-sites underscored)- **RBS*-sfGFPAAV-**B1006

gattcgttaccaattgacaGTAGCCTTTAGTCCATGtataatgctagctaACCTAGACCAAAGCCGGTAGTTAGTGGAGGTtactagATGCGTAAAGGCGAAGAGCTGTTCACTGGTGTCGTCCCTATTCTGGTGGAACTGGATGGTGATGTCAACGGTCATAAGTTTTCCGTGCGTGGCGAGGGTGAAGGTGACGCAACTAATGGTAAACTGACGCTGAAGTTCATCTGTACTACTGGTAAACTGCCGGTACCTTGGCCGACTCTGGTAACGACGCTGACTTATGGTGTTCAGTGCTTTGCTCGTTATCCGGACCATATGAAGCAGCATGACTTCTTCAAGTCCGCCATGCCGGAAGGCTATGTGCAGGAACGCACGATTTCCTTTAAGGATGACGGCACGTACAAAACGCGTGCGGAAGTGAAATTTGAAGGCGATACCCTGGTAAACCGCATTGAGCTGAAAGGCATTGACTTTAAAGAAGACGGCAATATCCTGGGCCATAAGCTGGAATACAATTTTAACAGCCACAATGTTTACATCACCGCCGATAAACAAAAAAATGGCATTAAAGCGAATTTTAAAATTCGCCACAACGTGGAGGATGGCAGCGTGCAGCTGGCTGATCACTACCAGCAAAACACTCCAATCGGTGATGGTCCTGTTCTGCTGCCAGACAATCACTATCTGAGCACGCAAAGCGTTCTGTCTAAAGATCCGAACGAGAAACGCGATCATATGGTTCTGCTGGAGTTCGTAACCGCAGCGGGCATCACGCATGGTATGGATGAACTGTACAAAGCTAGCGCAGCGAACGACGAAAATTACGCC**GCG**GCA**GTT**TAATAAaaaaaaaaaccccgcccctgacagggcggggtttttttt

EL222-expression construct

Ptrc1Ocore (*lac* operator underscored)-BCD2-**EL222-rnpB_T1**

GAGCTGTTGACAATTGTGAGCGCTCACAATATAATGTGTGGAAGGGCCCAAGTTCACTTAAAAAGGAGATCAACAATGAAAGCAATTTTCGTACTGAAACATCTTAATCATGCTAAGGAGGTTTTCTAATGGGTGCGGATGACACCCGCGTTGAGGTGCAACCTCCTGCGCAATGGGTTCTGGACCTGATCGAGGCCAGCCCTATCGCCAGTGTTGTGTCTGATCCTCGTCTGGCCGATAATCCGCTGATCGCCATTAATCAAGCCTTTACCGACCTGACCGGCTATAGCGAAGAAGAATGCGTGGGCCGCAATTGCCGTTTCCTGGCGGGTTCCGGCACCGAGCCTTGGCTGACCGACAAGATCCGCCAAGGCGTGCGCGAGCACAAGCCGGTGCTGGTTGAGATCCTGAACTACAAGAAGGACGGCACCCCTTTCCGCAATGCCGTGCTGGTTGCCCCGATCTACGATGACGACGACGAGCTGCTGTATTTCCTGGGCAGCCAGGTTGAAGTTGACGACGACCAGCCTAACATGGGCATGGCCCGCCGCGAACGCGCCGCCGAAATGCTGAAGACCCTGAGTCCTCGCCAGCTGGAGGTTACCACGTTAGTGGCCAGCGGCCTGCGCAACAAGGAAGTGGCGGCCCGCCTGGGCCTGTCCGAGAAAACCGTCAAGATGCACCGCGGCCTGGTGATGGAAAAGCTGAACCTGAAGACCAGCGCCGATCTGGTGCGCATTGCCGTGGAAGCCGGTATCTAATAAtcggtcagtttcacctgatttacgtaaaaacccgcttcggcgggtttttgcttttggaggggcagaaagatgaatgactgtc

Flag-EL222-expression construct

Ptrc1Ocore (*lac* operator underscored)-BCD2-FLAGtag-**EL222-rnpB_T1**

GAGCTGTTGACAATTGTGAGCGCTCACAATATAATGTGTGGAAGGGCCCAAGTTCACTTAAAAAGGAGATCAACAATGAAAGCAATTTTCGTACTGAAACATCTTAATCATGCTAAGGAGGTTTTCTAATGGACTACAAAGACGATGATGACAAGGGTGCGGATGACACCCGCGTTGAGGTGCAACCTCCTGCGCAATGGGTTCTGGACCTGATCGAGGCCAGCCCTATCGCCAGTGTTGTGTCTGATCCTCGTCTGGCCGATAATCCGCTGATCGCCATTAATCAAGCCTTTACCGACCTGACCGGCTATAGCGAAGAAGAATGCGTGGGCCGCAATTGCCGTTTCCTGGCGGGTTCCGGCACCGAGCCTTGGCTGACCGACAAGATCCGCCAAGGCGTGCGCGAGCACAAGCCGGTGCTGGTTGAGATCCTGAACTACAAGAAGGACGGCACCCCTTTCCGCAATGCCGTGCTGGTTGCCCCGATCTACGATGACGACGACGAGCTGCTGTATTTCCTGGGCAGCCAGGTTGAAGTTGACGACGACCAGCCTAACATGGGCATGGCCCGCCGCGAACGCGCCGCCGAAATGCTGAAGACCCTGAGTCCTCGCCAGCTGGAGGTTACCACGTTAGTGGCCAGCGGCCTGCGCAACAAGGAAGTGGCGGCCCGCCTGGGCCTGTCCGAGAAAACCGTCAAGATGCACCGCGGCCTGGTGATGGAAAAGCTGAACCTGAAGACCAGCGCCGATCTGGTGCGCATTGCCGTGGAAGCCGGTATCTAATAAtcggtcagtttcacctgatttacgtaaaaacccgcttcggcgggtttttgcttttggaggggcagaaagatgaatgactgtc

Combined P*EL* reporter and Flag-EL222-expression constructs

P*EL*(EL222 binding-sites underscored)- **RBS*-sfGFPAAV-**B1006-Ptrc1Ocore (*lac* operator underscored)-BCD2-FLAGtag-**EL222-rnpB_T1**

gattcgttaccaattgacaGTAGCCTTTAGTCCATGtataatgctagctaACCTAGACCAAAGCCGGTAGTTAGTGGAGGTtactagATGCGTAAAGGCGAAGAGCTGTTCACTGGTGTCGTCCCTATTCTGGTGGAACTGGATGGTGATGTCAACGGTCATAAGTTTTCCGTGCGTGGCGAGGGTGAAGGTGACGCAACTAATGGTAAACTGACGCTGAAGTTCATCTGTACTACTGGTAAACTGCCGGTACCTTGGCCGACTCTGGTAACGACGCTGACTTATGGTGTTCAGTGCTTTGCTCGTTATCCGGACCATATGAAGCAGCATGACTTCTTCAAGTCCGCCATGCCGGAAGGCTATGTGCAGGAACGCACGATTTCCTTTAAGGATGACGGCACGTACAAAACGCGTGCGGAAGTGAAATTTGAAGGCGATACCCTGGTAAACCGCATTGAGCTGAAAGGCATTGACTTTAAAGAAGACGGCAATATCCTGGGCCATAAGCTGGAATACAATTTTAACAGCCACAATGTTTACATCACCGCCGATAAACAAAAAAATGGCATTAAAGCGAATTTTAAAATTCGCCACAACGTGGAGGATGGCAGCGTGCAGCTGGCTGATCACTACCAGCAAAACACTCCAATCGGTGATGGTCCTGTTCTGCTGCCAGACAATCACTATCTGAGCACGCAAAGCGTTCTGTCTAAAGATCCGAACGAGAAACGCGATCATATGGTTCTGCTGGAGTTCGTAACCGCAGCGGGCATCACGCATGGTATGGATGAACTGTACAAAGCTAGCGCAGCGAACGACGAAAATTACGCC**GCG**GCA**GTT**TAATAAaaaaaaaaaccccgcccctgacagggcggggttttttttTACTAGAGAGCTGTTGACAATTGTGAGCGCTCACAATATAATGTGTGGAAGGGCCCAAGTTCACTTAAAAAGGAGATCAACAATGAAAGCAATTTTCGTACTGAAACATCTTAATCATGCTAAGGAGGTTTTCTAATGGACTACAAAGACGATGATGACAAGGGTGCGGATGACACCCGCGTTGAGGTGCAACCTCCTGCGCAATGGGTTCTGGACCTGATCGAGGCCAGCCCTATCGCCAGTGTTGTGTCTGATCCTCGTCTGGCCGATAATCCGCTGATCGCCATTAATCAAGCCTTTACCGACCTGACCGGCTATAGCGAAGAAGAATGCGTGGGCCGCAATTGCCGTTTCCTGGCGGGTTCCGGCACCGAGCCTTGGCTGACCGACAAGATCCGCCAAGGCGTGCGCGAGCACAAGCCGGTGCTGGTTGAGATCCTGAACTACAAGAAGGACGGCACCCCTTTCCGCAATGCCGTGCTGGTTGCCCCGATCTACGATGACGACGACGAGCTGCTGTATTTCCTGGGCAGCCAGGTTGAAGTTGACGACGACCAGCCTAACATGGGCATGGCCCGCCGCGAACGCGCCGCCGAAATGCTGAAGACCCTGAGTCCTCGCCAGCTGGAGGTTACCACGTTAGTGGCCAGCGGCCTGCGCAACAAGGAAGTGGCGGCCCGCCTGGGCCTGTCCGAGAAAACCGTCAAGATGCACCGCGGCCTGGTGATGGAAAAGCTGAACCTGAAGACCAGCGCCGATCTGGTGCGCATTGCCGTGGAAGCCGGTATCTAATAAtcggtcagtttcacctgatttacgtaaaaacccgcttcggcgggtttttgcttttggaggggcagaaagatgaatgactgtc

**Supplementary figures**


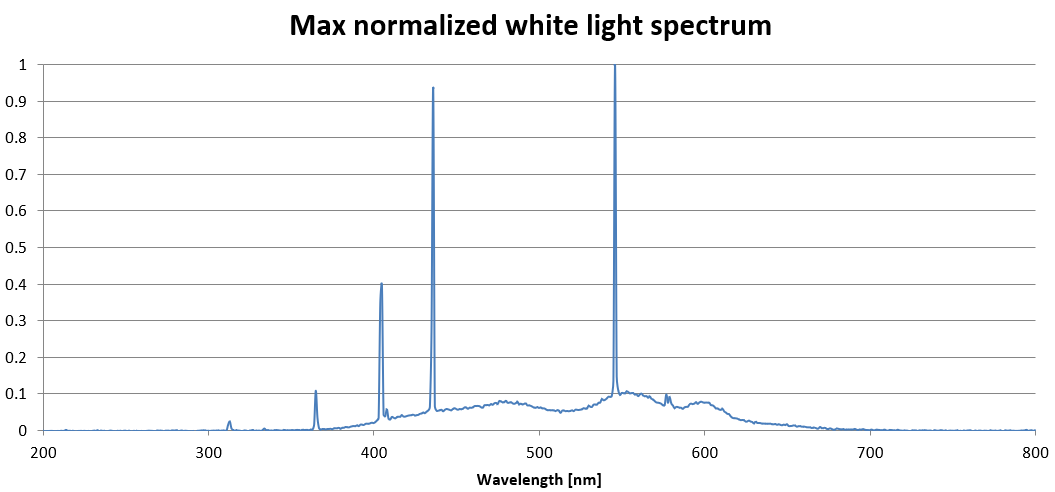


**Figure S1.** Spectrum of white light normalized to maximum photon counts. The underlying data is available in Data file S3.


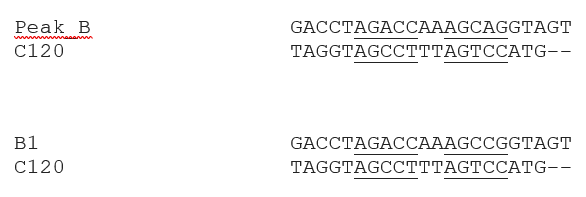


**Figure S2.** Alignment of the C120 operator with the peak Band B1 operators. The EL222-binding site motifs are underscored.

**Supplementary tables**

| **Name** | **Sequence, 5’ to 3’** |
| --- | --- |
| P(EL119CP)-sfGFPaav-f1 | atgaattctctagagattcgttaccaattgacagtagcctttagtccatgtataatgctagctaacctagaccaaagccggtagttagtggaggttactagatgcgtaaaggcgaagagc |
| aav-B1006-r1 | gaactgcagactagtaaaaaaaaaccccgccctgtcaggggcggggtttttttttttattaaactgccgcggcgtaattttcgtcgttcg |
| PLlacSyn-EL222-f1 | aatgaattctctagaataaatgtgagcggataacattgacattgtgagcggataacaatataatgcacactactagagtagtggaggttactagatgggtg |
| rnpBT1-r1 | tagctgcagactagtagacagtcattcatctttctgcccctccaaaagcaaaaacccgccgaag |
| P1OcBCD2EL-f1 | agttcacttaaaaaggagatcaacaatgaaagcaattttcgtactgaaacatcttaatcatgctaaggaggttttctaatgggtgcggatgacaccc |
| P1OcBCD2FlgEL-f1 | ttcacttaaaaaggagatcaacaatgaaagcaattttcgtactgaaacatcttaatcatgctaaggaggttttctaatggactacaaagacgatgatgacaagggtgcggatgacaccc |
| X-P1OcBCD2-f2 | ttttctagagagctgttgacaattgtgagcgctcacaatataatgtgtggaagggcccaagttcacttaaaaaggagatcaac |

**Table S1**. DNA oligonucleotide sequences.

**Supplementary data files**

Data file S1: Plate reader data from the characterization of EL222 expression & fluorescence.

Data file S2: The EL222 fluorescence emission data.

Data file S3: Data of white light used for EL222 repression assays.

Data file S4: Plate reader data and data analysis from the time-course characterization of light-regulated EL222-mediated P*_EL_* repression.
